# Feasibility of an MR-based digital specimen for tongue cancer resection specimens: a novel approach for margin evaluation

**DOI:** 10.3389/fonc.2024.1342857

**Published:** 2024-03-28

**Authors:** Klijs Jacob de Koning, Jan Willem Dankbaar, Bart de Keizer, Koen Willemsen, Annette van der Toorn, Gerben Eise Breimer, Robert Jelle Johan van Es, Remco de Bree, Rob Noorlag, Marielle Emile Petronella Philippens

**Affiliations:** ^1^ Department of Head and Neck Surgical Oncology, University Medical Center Utrecht, Utrecht, Netherlands; ^2^ Department of Radiology, University Medical Center Utrecht, Utrecht, Netherlands; ^3^ 3D Lab, University Medical Center Utrecht, Utrecht, Netherlands; ^4^ Translational Neuroimaging Group, Center for Image Sciences, University Medical Center Utrecht & Utrecht University, Utrecht, Netherlands; ^5^ Department of Pathology, University Medical Center Utrecht, Utrecht, Netherlands; ^6^ Department of Radiotherapy, University Medical Center Utrecht, Utrecht, Netherlands

**Keywords:** tongue cancer, oral cancer, resection margin, magnetic resonance imaging, image guided surgery, high-field

## Abstract

**Objective:**

This study explores the feasibility of ex-vivo high-field magnetic resonance (MR) imaging to create digital a three-dimensional (3D) representations of tongue cancer specimens, referred to as the “MR-based digital specimen” (MR-DS). The aim was to create a method to assist surgeons in identifying and localizing inadequate resection margins during surgery, a critical factor in achieving locoregional control.

**Methods:**

Fresh resection specimens of nine tongue cancer patients were imaged in a 7 Tesla small-bore MR, using a high-resolution multislice and 3D T2-weighted Turbo Spin Echo. Two independent radiologists (R1 and R2) outlined the tumor and mucosa on the MR-images whereafter the outlines were configured to an MR-DS. A color map was projected on the MR-DS, mapping the inadequate margins according to R1 and R2. We compared the hematoxylin-eosin-based digital specimen (HE-DS), which is a histopathological 3D representation derived from HE stained sections, with its corresponding MR-images. In line with conventional histopathological assessment, all digital specimens were divided into five anatomical regions (anterior, posterior, craniomedial, caudolateral and deep central). Over- and underestimation 95^th^-percentile Hausdorff-distances were calculated between the radiologist- and histopathologist-determined tumor outlines. The MR-DS’ diagnostic accuracy for inadequate margin detection (i.e. sensitivity and specificity) was determined in two ways: with conventional histopathology and HE-DS as reference.

**Results:**

Using conventional histopathology as a reference, R1 achieved 77% sensitivity and 50% specificity, while R2 achieved 65% sensitivity and 57% specificity. When referencing to the HE-DS, R1 achieved 94% sensitivity and 61% specificity, while R2 achieved 88% sensitivity and 71% specificity. Range of over- and underestimation 95HD was 0.9 mm - 11.8 mm and 0.0 mm - 5.3 mm, respectively.

**Conclusion:**

This proof of concept for volumetric assessment of resection margins using MR-DSs, demonstrates promising potential for further development. Overall, sensitivity is higher than specificity for inadequate margin detection, because of the radiologist’s tendency to overestimate tumor size.

## Introduction

An estimated 3.5 new cases of tongue cancer occur annually in the United States for every 100,000 individuals. Squamous cell carcinoma of the tongue is the most predominant type of oral cancer (1). Complete surgical removal of the primary tumor is the first treatment choice to prevent local recurrence (2).

After surgical excision, the margins of the resection specimen are analyzed during histopathological examination to verify that the minimal margin distance is adequate (≥5 mm) or inadequate (<5 mm) (3). Unfortunately, inadequate margins are frequently encountered (4). A retrospective analysis of 96 SCCT patients treated at our center revealed that 84% of the resection specimens had inadequate margins (5), which is in line with the literature (4). These patients may be considered as candidates for local adjuvant treatment, (chemo)radiotherapy or secondary resection. Local radiotherapy has several side effects, including mucositis, xerostomia, and osteoradionecrosis (6, 7). Furthermore, conducting secondary surgery not only requires additional operating time and anesthesia but it also introduces uncertainty regarding the anatomical relationship between the newly obtained resection specimen and the original specimen (8).

By assessing margins intra-operatively, surgeons can make immediate adjustments, eliminating the drawbacks of a second surgery. Several techniques exist for assessing margins: either during the resection (in-vivo) (5, 9–14) or immediately after (ex-vivo) (5, 12, 15–19). Frozen section analysis is the most frequently used ex-vivo method. However, it is prone to sampling errors as only a small portion of the resection specimen and/or wound bed is sampled (20). Further complicating this is the challenge in linking the frozen section sample to the resection specimens (21). Over the past decade, several publications have addressed using magnetic resonance (MR) for intra-operative margin assessment (22–24). While only a few institutions have a clinical MR-machine in the operating room, a high-field small-bore MR, typically located outside the operating room, produces high-quality images (23). Despite the inability to assess margins in-vivo, MR has the potential to generate three-dimensional (3D) representations of the resection specimen. The fact that such a 3D representation allows examiners to view the resection specimen from multiple angles and perspectives, contrasts with the small sampling rate of frozen section analysis.

This study serves as a proof of concept for using a high-field ex-vivo MR as an alternative to frozen section analysis for improved localization of inadequate margins. Digital MR-based 3D representations of the specimen, mapping the inadequate margins, were validated in two ways: 1) with conventional histopathological assessment as reference and 2) with a 3D representation of the specimen based on histopathology.

## Methods

### MR-image acquisition and qualitative assessment

**Figure 1:**
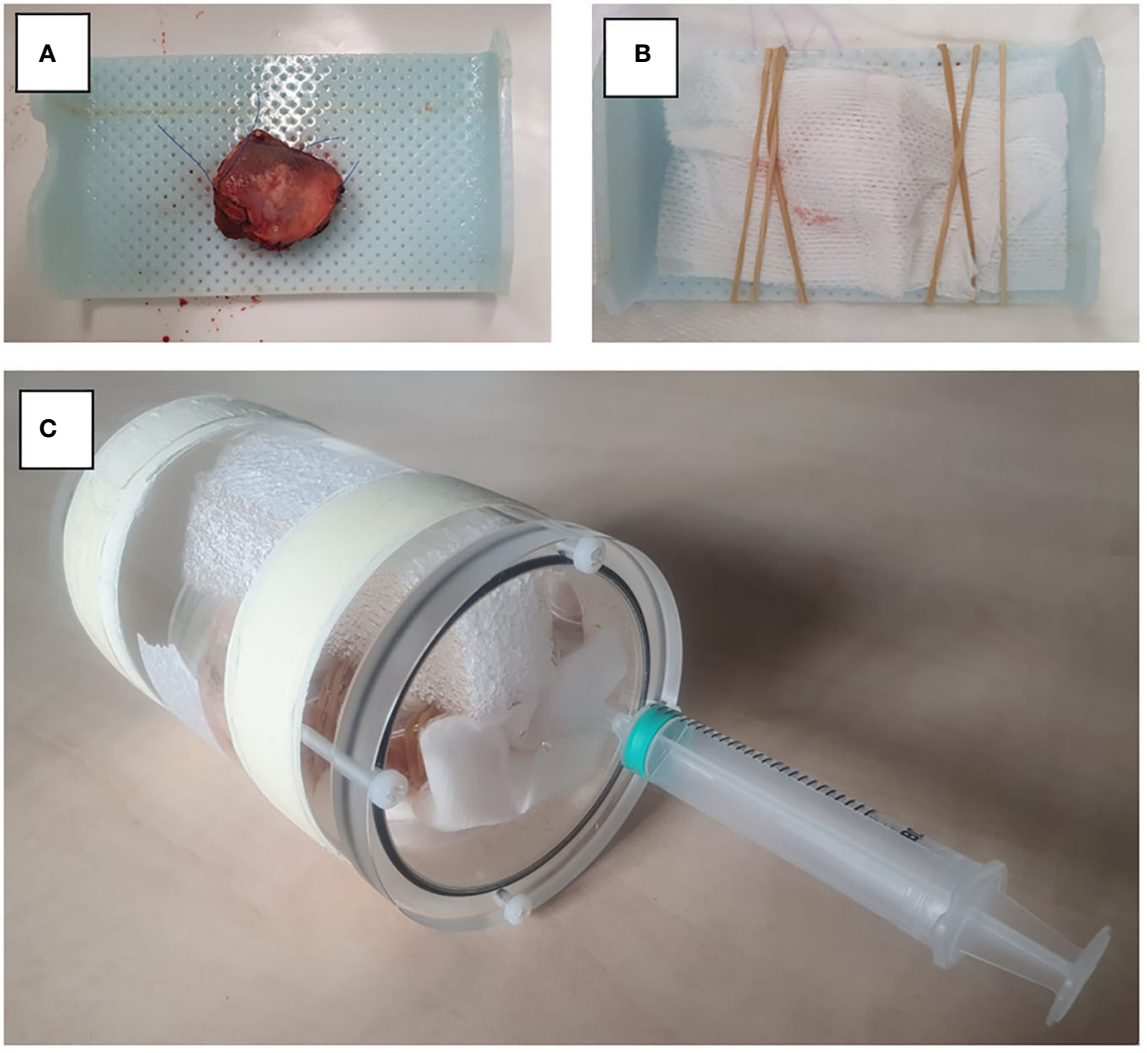
Set-up for MR image acquisition. **(A)** Resection specimen is placed on thermoplastic material. **(B)** Fixation of the specimen with gauze. **(C)** Resection specimen is placed in a PMMA cylindrical container.

Nine patients who underwent surgery for cT1-T3 squamous cell carcinoma of the tongue were prospectively included between January and June 2021. Sutures were applied to the specimen to facilitate orientation during scanning and pathological examination. Directly after surgery, the fresh resection specimens were transported to a small-bore 7T MR-machine (BioSpec 7T, Bruker, Ettlingen, Germany), with a 0.9 T/m gradient system, interfaced with a Philips console (Philips Medical Systems, Best, Netherlands R.5.4). Additional re-resections were not analysed. The resection specimens were placed on a support made of thermoplastic material and were fixated with a gauze. On this support, the resection specimens were placed in a poly-methyl methacrylatecylindrical container (outer diameter: 70mm, inner diameter: 59mm). The container was filled with perfluoropolyether fluorinated fluid (Galden, Solvay Solexis, Thorofare, NJ, USA) to prevent susceptibility artifacts during scanning. The container was placed in a transmit-receive volume coil with a 72 mm inner diameter and 112 mm outer diameter (Bruker) (**Figure 1**). The B0-field homogeneity was enhanced with shimming up to the second order. For each case, four scanning sequences were used: a 3D T2-weighted (T2W) Turbo Spin Echo (TSE) with an isotropic voxel size of 0.3 mm^3^ (referred to as MR 3D-images) and a T2W TSE with an in-plane resolution of 0.125 mm^2^ and 1.0 mm slice thickness in three orthogonal directions (referred as MR multislice-images). Details of these sequences are provided in **Table 1**. Two radiologists assessed image quality by independently rating the images using a 5-point Likert scale on four parameters: 1) overall image quality, 2) visibility of the tumor, 3) visibility of the transition between mucosa and resection plane, and 4) certainty of margin status.

**Table 1:** MRI acquisition parameters.

Sequence name	Scan direction	TR (ms range)	TE (ms)	RA (°)	Slice Thickness (mm, range)	Gap distance (µm)	No. Slices (range)	Acquired Voxel size (mm^2^)	Scanning time (min., range)	Echo train
T2W TSE Multislice	Sagittal	8554-17412	80	140	9-10	0.10	32-51	0.125	4:51-12:34	13
T2W TSE Multislice	Axial	6415- 21066	80	140	10	0.10	23-27	0.125	5:43 -11:02	13
T2W TSE Multislice	Coronal	10692- 22906	80	140	10	0.10	17-55	0.125	5:06-17:56	13
T2W TSE 3D	Coronal	2000	140	60	0.25-0.30	–	100-140	0.25 -0.30	10:45-36:06	32

T2W, T2 weighted signal; TSE, Turbo Spin Echo; 3D, three-dimensional; TR, Repetition time; TE, Echo time; RA, Refocusing Angle.

### Histopathological assessment

**Figure 2:**
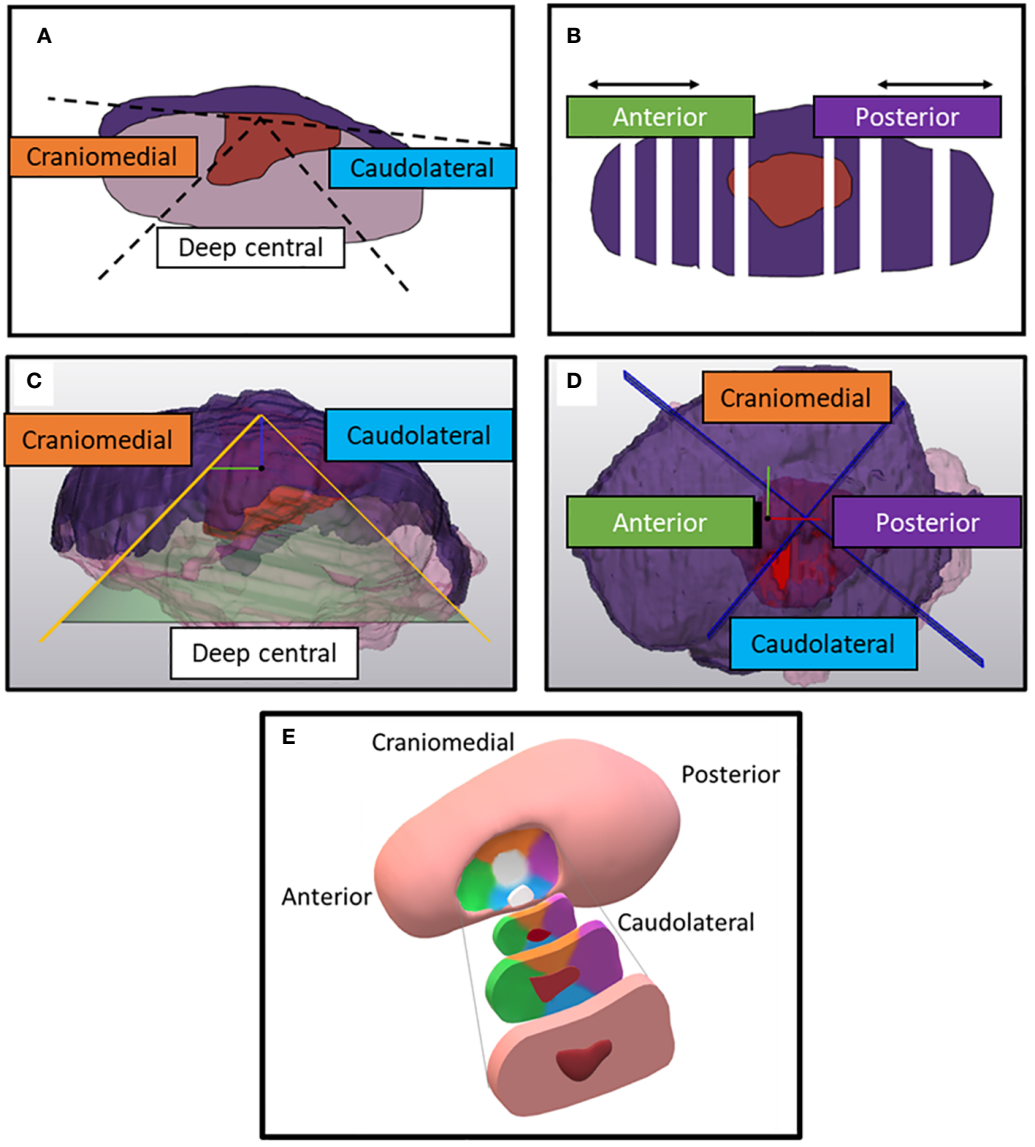
Division of the specimen on histopathological HE-sections (according to conventional histopathological assessment) and on the digital specimens. **(A)** Two 45-degree lines diverge from a line parallel to the mucosa when a HE-section is faced from the front. In between the 45-degree lines, the specimen’s portion is defined as deep central. Outside these two 45-degree lines, the specimen's portion is either defined as craniomedial (towards the dorsum of the tongue) or caudolateral (towards the floor of mouth). **(B)** Top view of the specimen. Anterior and posterior margins are determined by multiplying the number of tumor-free slices with mean thickness of the tissue blocks. **(C)** Side view of the digital specimen which is divided by a conus from of which the apex is perpendicular to a plane fitted on the mucosa. The inclination of the conus is 45-degree. **(D)** Top view of the remaining portion of the digital specimen. This portion is divided by two perpendicular planes in four quadrants. The reference system for those planes is created perpendicular to the base of the cone and combined with a manually selected anterior and posterior point. **(E)** Anterior, posterior, craniomedial, caudolateral, deep central location of the resection specimen, depicted in a schematic figure of the tongue. The color of the specimen’s location corresponds with the colors in the text boxes of figure **(A–D)**.

After MR-imaging, the fresh resection specimens underwent fixation in a 4% formaldehyde solution for a minimum of 24 hours before histopathological examination. The specimens were sliced into cross-sectional tissue blocks of approximately 3 to 5 mm thick, oriented perpendicular to the anterior-posterior axis. From each section, a 4 μm thick microscopic section was obtained and stained with hematoxylin and eosin (HE). After staining, the sections were converted to digital images (referred to as HE-images) using the methods of Stathonikos et al. (25). The margins of the resection specimens were determined at five specific locations: anterior, posterior, craniomedial (towards the dorsal surface of the tongue), caudolateral (towards the floor of mouth), and deep central (directly under the tumor). The anterior and posterior margins were determined by multiplying the average thickness of a single slice (derived from the specimen´s length divided by the number of slices taken) with the count of microscopically tumor-free slices in the respective anterior and posterior directions. The craniomedial and caudolateral locations were defined as the space between two 45-degree lines originating from a line parallel to the mucosa through the middle of the tumor. The deep central location encompassed the region between the craniomedial and caudolateral slices. Our center’s department of pathology adopted this method to distinguish the margins of the five locations, introduced during one of our previous studies (**Figures 2A, B**) (5). The margins and tumor thickness were measured using a digital ruler within dedicated software to assess microscopic images (Sectra IDS7, version 23.1, Linkoping, Sweden).

### Creation of the MR-based digital specimen and HE-based digital specimen

#### Registration

**Figure 3:**
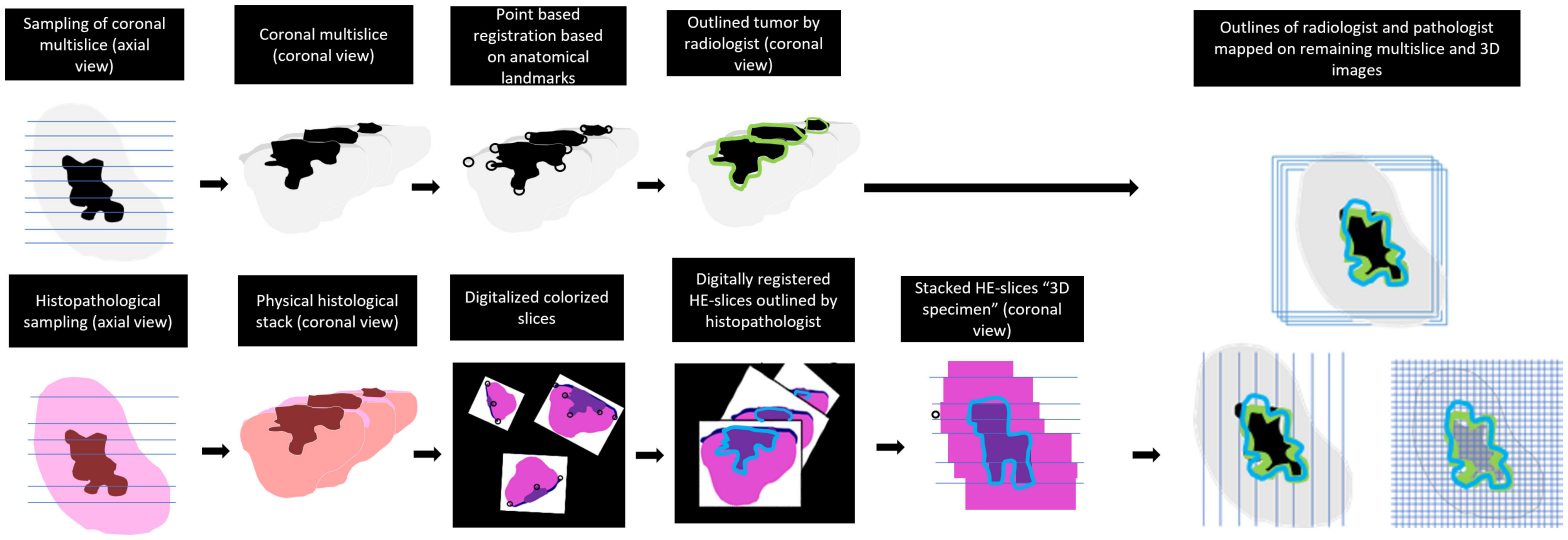
Workflow of the registration and mapping of the 3D outlines of MR-images on all obtained sequences.

**Figure 4:**
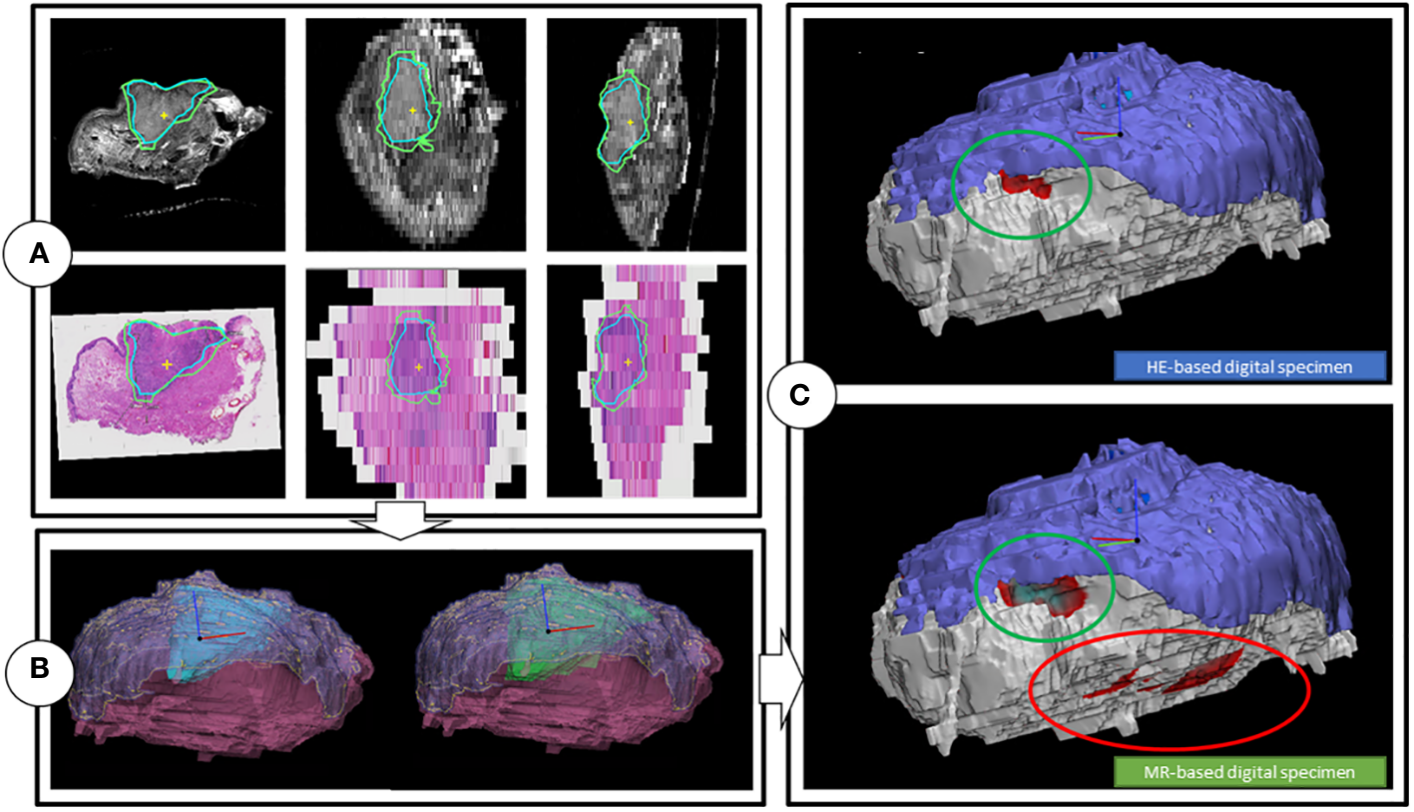
Workflow of the creation of the digital specimens. **(A)** Coronal, transversal and sagittal view of the T2W multislice MR-images, while matched with the HE-images. Includes tumor outlines by a histopathologist (blue line) and a outline of one of the two radiologists (green). **(B)** HE-DS (left) and MR-DS (right). Note that the representations of the tumors are different as the one of the HE-DS (blue) is derived from the pathologist’s outline and the one of the MR-DS (green) is derived from the radiologist’s outline. The mucosa (purple) and specimen’s outline (pink), representing the resection plane are visible as well. **(C)** Colormaps projected on the HE-DS (upper) and MR-DS (lower), highlighting the regions with inadequate margins in dark red, according to respectively histopathology and radiology. Green ellipsoid is a true positive inadequate margin. Red ellipsoid is a false positive inadequate margin. For the sake of clarity, the resection plane is represented in white. The mucosa is represented in purple.

**Figure 5:**
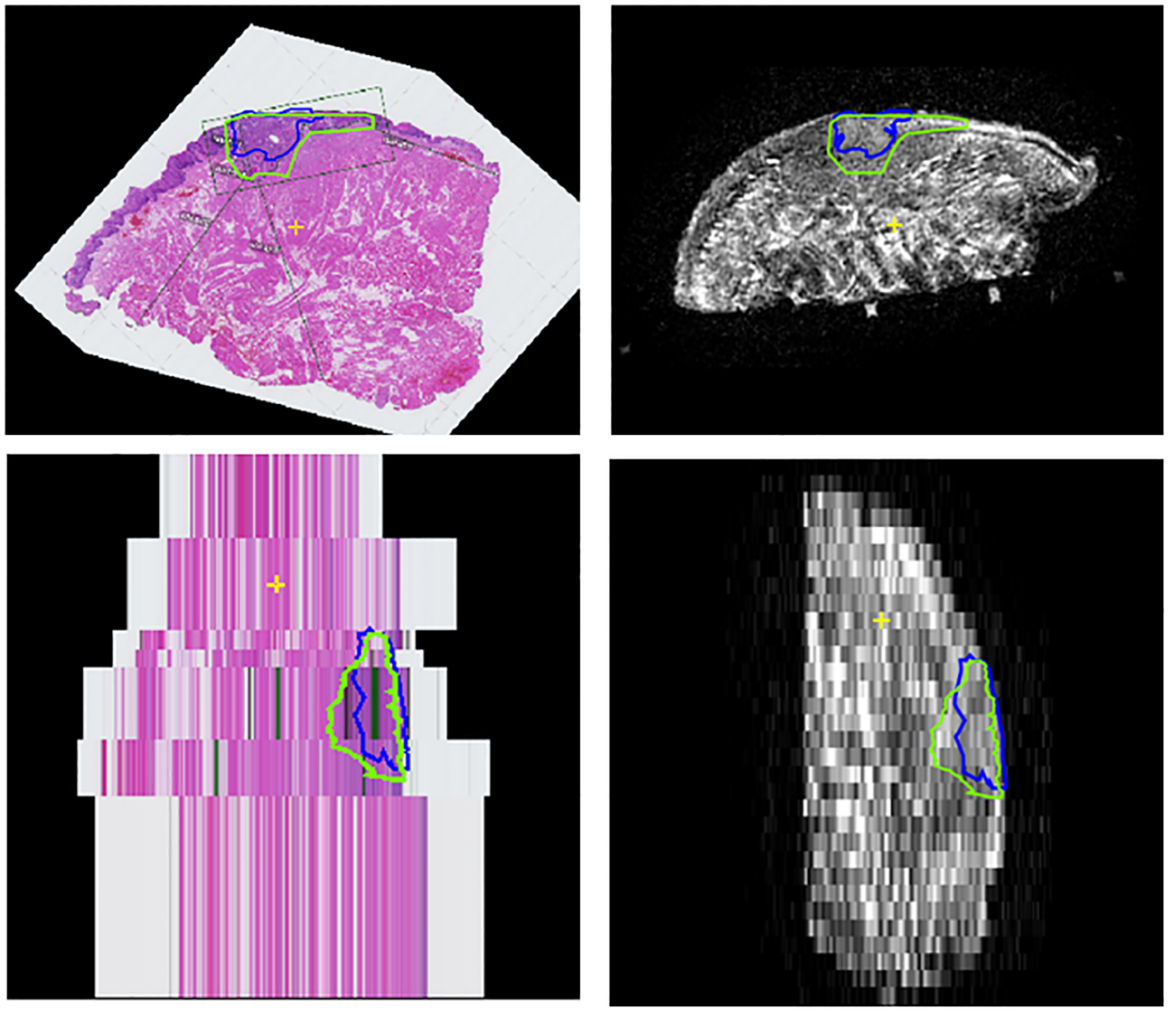
Coronal and sagittal views of the stacked HE-stained images (left column) and coronal T2W MR-image (right column) of case 8. As can be seen in this case, the HE-stained image spacing can be irregular and may contribute to underestimation of the anterior and posterior margin in the histopathological report; in the clinical report the anterior margin (lower slice) has been denoted as inadequate, since only one HE-stained image was tumor free and the main cross-section thickness was determined as 4.3 mm. However, after matching the HE-stained images with the coronal T2W MR-images, this distance seemed to be far larger.

The MR- and HE-images were imported in in-house built viewing and contouring software (Volumetool, version 1.30.39) (26). The coronal T2W images were used for registration with the HE-images. The choice of using this particular sequence had two reasons. Firstly, tumors exhibit better contrast with normal tissue in T2W multi-slice MR images than in T2W MR 3D-images. This is because in a 3D T2W TSE-scan small refocusing angles need to be used to preserve signal and prevent blurring due to T2 decay. Therefore, the T2 contrast is different from a multi slice T2W image. Secondly, the orientation of the coronal plane aligns with our institution’s recommended approach for resection specimen slicing to obtain HE-sections, which is specifically perpendicular to the anterior-posterior axis. Matching pairs of T2W slices and HE-images were selected. A point-based registration technique was used, where two observers (KK and JR) selected corresponding anatomical points (e.g., mucosa, tumor-protrusions, and arteries). Subsequently, the rotated and scaled HE-images were digitally stacked. This resulted in a volumetric representation of the histopathological situation, comparable to a coronal MR dataset. The observers could also adjust the distance between each HE-image within the specimen (**Figures 3**, **4A**, **5**). This procedure is similar to the methods described in the work of Caldas Magalhaes et al. (27).

#### Outline procedure

The radiologists contoured the tumor and mucosa by outlining both structures on the coronal T2W images. The pathologist was asked to perform the same procedure on the HE-images. This resulted in a volumetric outline for both the tumour and the mucosa, each determined independently by radiologists and a pathologist (**Figures 3**, **4A**). Pathologist-determined volumetric outlines were propagated to the available MR sequences and slightly modified by three authors (KK, JR and MP) until mutual agreement was achieved. These modifications were considered justifiable because the relatively large variance of the HE-images’ spacing (1-10 mm) and because histopathological processing may affect the accuracy of the HE-images (i.e. shrinkage, tissue deformation, missing tissue) (**Figure 5**). Radiologist-determined volumetric outlines were propagated to the other MR-sequences (i.e. sagittal, transversal and 3D images) and to the stacked HE-images. In this way, the volumetric outlines of the radiologist could be compared with those of the pathologist (**Figures 3**, **4A**). Using Volumetool, a threshold was applied to distinguish the background from the specimen on the images of the MR 3D-sequence to obtain a volumetric outline of the specimen’s contour.

#### MR-based and HE-image based digital specimens

Using medical image processing software, Mimics (v24.0, Materialise, Leuven, Belgium) and computer-assisted-design and modelling software 3-matic (v17.0, Materialise, Leuven, Belgium) the volumetric outline of the tumor, mucosa and specimen’s outer contour were combined to attain a 3D visual representation of the resection specimen; two as determined by the independent radiologists, referred to as the MR-based digital specimen (MR-DS) and one as determined by the independent pathologist, referred to as the HE-based digital specimen (HE-DS) (**Figure 4B**). The outer contour of the HE-DS was obtained from the MR-images to compensate for the artifacts that occur during histopathological processing.

For comparison purposes, a 3D substitute to the aforementioned conventional assessment of the five margin directions i.e. anterior, posterior, craniomedial, caudolateral and deep central was created (5) (**Figure 2**). Firstly, a cone with an apex angle of 45 degrees was created to designate the deep central region of the MR and HE-DS. The apex of the cone was placed manually in the middle of the tumor with its base perpendicular to a plane fitted onto the mucosa. Secondly, the portion of the MR- or HE-DS outside of the cone was divided into 4 quadrants. To define the quadrants, an anterior-posterior midplane was created perpendicular to the cone base by manually selecting an anterior and posterior point. This midplane was used as a reference system to create two perpendicular planes and divide the remaining portion of the MR- or HE-DS into the anterior, posterior, craniomedial and caudolateral quadrants (**Figure 2**). By applying a distance colormap on the MR- or HE-DS outline, highlighting regions were the distance between tumor and specimen’s outline was <5 mm, the MR- and HE-DS could be used to localize inadequate margins according to radiologist-determined outline on MR-images and pathologist-determined outline on HE-images (**Figure 4C**).

### Analyses

Statistical analysis was performed using SPSS (version 27.0, IBM, Armonk, NY, USA).

To assess the accuracy of the radiologist-determined volumetric outlines of the tumor, when compared to its histopathologic counterpart, we utilized the 95th percentile Hausdorff distance (95HD). This statistic measures the maximum distance between corresponding points between the MR- and HE-DS’ tumors, while accounting for data variability and outliers. It was calculated in two ways:

- Underestimation 95HD: We computed the 95HD between the pathologist’s tumor outline and the Boolean intersection with the radiologists’, revealing tumor underestimation by the MR-DS.
- Overestimation 95HD: We computed the 95HD between the radiologists’ tumor outline and the Boolean intersection with the pathologist’s, revealing tumor overestimation by the MR-DS.

Furthermore, the accuracy of the MR-DS in inadequate margin (i.e. <5 mm) prediction was assessed per location (e.g., anterior, posterior, craniomedial, caudolateral and deep) in two manners: 1) by comparing it with the margins of all five locations, as described by the conventional method of histopathological margin assessment and 2) by comparing it with the HE-DS. It was determined whether the location of the highlighted regions that indicated <5 mm on the MR-DS corresponded with those on HE-DS (**Figures 2** and **4**). Accuracy was presented as sensitivity, specificity, positive predictive value (PPV), and negative predictive value (NPV).

As the conventional histopathological margin assessment is the gold standard, and not the HE-DS, we compared location of the inadequate margins according to the HE-DS with the results of conventional pathology. This allowed us to explain differences in diagnostic accuracy when using the different reference standards.

## Results

### Clinical characteristics

The cohort consisted of 9 cases with various tumor stages: pT1 (4 cases), pT2 (2 cases), and pT3 (3 cases). The mean (SD) of depth of invasion (DOI) was 6.9 (4.9) mm. Unfavorable histopathological growth patterns (i.e. non-cohesive growth, perineural growth, and vascular invasion) were noted as follows: none for two cases, 1 for three cases, 2 for three cases, and 3 for one case. All cases exhibited moderately differentiated tumors based on histopathology, except for case 4, which had a well-differentiated tumor (**Table 2**).

**Table 2:** Case-specific characteristics.

#	Tumor characteristics							95^th^ percentile Hausdorff distance of the tumor			
	T- stage	DOI	Max. diameter	No. HGP	Differentiation	Margin status	Min. margin conv. histo-pathology	U95HD R1 (mm)	O95HD R1 (mm)	U95HD R2 (mm)	O95HD R2 (mm)
1	pT3	15.6	44	3	±	I	1.9	1.7	0.9	1.2	1.6
2	pT3	10.0	27	0	±	I	2.5	0.7	2.5	1.5	1.8
3	pT3	12.6	28	2	±	I	1.0	1.3	6.4	0.3	5.2
4	pT1	1.2	3	0	+	A	6.7	0.5	10.0	1.4	6.6
5	pT2	7.0	7	2	±	I	3.2	0.7	0.6	0.3	1.6
6	pT1	2.4	10	1	±	I	4.5	0.9	3.1	0.4	5.5
7	pT2	6.7	30	2	±	I	2.3	0.9	1.2	0.4	1.2
8	pT1	3.5	15	1	±	I	4.3	0.9	2.0	0.5	3.1
9	pT1	3.4	15	1	±	I	4.6	0.0	11.8	5.3	2.5

T-stage, Tumor stage; DOI, Depth of invasion; No. HGF, Number of unfavorable histopathological growth factors; Max. diameter, Maximal diameter; Min. margin histopath; Minimal margin histopathology, U95HD, 95^th^ percentile Hausdorff distance of underestimation; O95HD, 95^th^ percentile Hausdorff distance of overestimation; R1, radiologist 1; R2, radiologist 2; ±, Moderately differentiated; + well differentiated; I, inadequate; A, adequate.

### Qualitative assessment of original ex-vivo MR-images

On a scale from 1 to 5, the median image quality was rated 4 (range: 3-4) by R1 and 3 (range: 3-5) by R2. The median visibility of the tumor was rated 4 (range: 2-4, case 3 received a score of 2) by R1 and 4 (range: 3-5) by R2. The visibility of the transition from the mucosa to the resection plane was rated 3 (range: 1-4, case 3 received a score of 1, and case 5 a score of 2) by R1 and 4 (range: 4-5) by R2. For image quality, both observers agreed with a maximum of 1 point difference in 100% of the cases. For tumor visibility this was 100% and 66%, respectively.

### Diagnostic accuracy of the MR-based digital specimens

**Figure 6:**
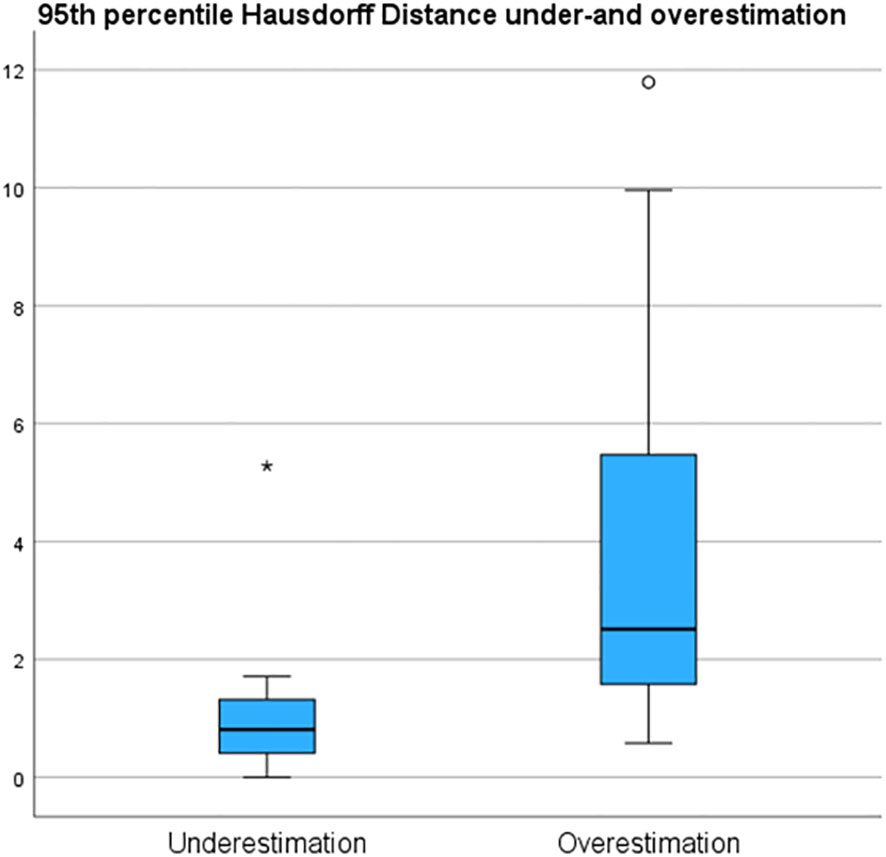
95^th^ percentile Hausdorff under and overestimation of the tumor margin in de MRI of the specimen (MR-DS)- with respect to the of the tumor in the HE-based digital specimen (HE-DS).

Underestimation 95HD and overestimation 95HD of the tumor extension as derived by the radiologists in MR-DSs are depicted in **Table 2** and **Figure 6**. Range of overestimation 95HD (0.9 mm – 11.8 mm) was far larger than the range of underestimation 95D (0.0 mm – 5.3 mm). Except for the outlier of 5.3 mm, the maximal underestimation 95HD was 1.7 mm. When comparing the MR-DS with conventional histopathological assessment, R1 exhibited higher sensitivity than R2, but lower specificity. This observation remained consistent when the MR-DSs were compared with the HE-DSs. In overall, the diagnostic accuracy was higher when referred to the HE-DS than when referred to conventional histopathological assesment (**Table 3**).

**Table 3:** Diagnostic accuracy of detection of inadequate margins (<5 mm).

Reference	Conventional histopathological assessment		MR-based digital specimen	
Radiologist	Radiologist 1	Radiologist 2	Radiologist 1	Radiologist 2
**Sensitivity (95% CI)**	77% (50% - 93%)	65% (38% - 86%)	94% (71% - 100%)	88% (64% -99%)
**Specificity (95% CI)**	50% (31% - 69%)	57% (37% - 76%)	61% (41% - 79%)	71% (51% - 87%)
**PPV (95% CI)**	48% (29% - 68%)	48% (27% - 69%)	59% (39% - 78%)	65% (43% - 84%)
**NPV (95% CI)**	78% (52% - 94%)	74% (50% - 89%)	94% (73% - 100%)	91% (71% - 99%)

### Comparison between conventional histopathology and HE-based digital specimens

To assess the impact of the HE-DS as a 3D reference, we conducted a comparison of its margins with those obtained through conventional histopathologic assesment. Of the forty-five histopathological margins measured with conventional histopathological assessment, nine (20%) were inconsistent with the HE-DS. For five of these inconsistencies, it became evident that the subdivision of the HE-DS into the anterior, posterior, caudolateral, craniomedial, and deep central regions did not correspond to the conventional subdivision (**Figures 2** and **4C**). Consequently, four regions in three cases were classified as adequate (≥ 5mm) by the conventional assessment and inadequate (<5 mm) by the HE-DS: anterior in case 1, anterior and posterior in case 3, and craniomedial in case 5. In the deep central part of case 5, the margin was classified as inadequate by the conventional assessment and adequate by the HE-DS. In three of the nine inconsistencies, the histopathological margin appeared slightly modified due to deformation during processing of the MR-or HE-images. This caused the deep central margin in the HE-DS for case 1 to be classified as inadequate due to compression during scanning. Two margins in two cases, i.e. case 2 and case 9, were classified as adequate by the HE-DS, but inadequate by the conventional method due to rupture and shrinkage during histopathological processing. As we used the specimen’s outline, derived from the MR-images, to compensate for these artifacts, the HE-DS’s margin differed from conventional margin assessment. In the last inconsistency, the thickness of both the most anterior and posterior HE-images of case 8 appeared to be greater than what was estimated by conventional assessment (**Figure 5**). As only these slices were tumor-free, the anterior and posterior margins were reported as inadequate, whereas, according to the HE-DS, they were judged adequate.

## Discussion

In this study, we evaluated whether the volumetric assessment of resection margins using MR-DSs, as determined by radiologists, could improve the localization of inadequate (<5 mm) margins. We compared the inadequate margins according to the MR-DS with 1) conventional histopathologic assessment and 2) with a HE-DS. When conventional histopathological margin assessment was used as a reference, we found that the MR-DS had moderate sensitivity and specificity for inadequate margin detection. However, when compared with a HE-DS, sensitivity and specificity were higher. Generally, the NPV for detecting inadequate margins is higher than the PPV. Apparently, the tumor’s volume is more often overestimation and underestimation. As the incidence of inadequate margins is relatively high, there is a relatively high frequency of false positives and a low frequency of false negatives, which results in a lower specificity but higher sensitivity.

There are several reasons why the diagnostic accuracy of MR-DS, when referred to HE-DS, was higher than when referred to conventional histological assessment. Firstly, the framework for the localization of inadequate margins on MR-DS (i.e. determining whether there was a region of inadequate margin at the anterior, posterior, craniomedial, caudolateral and deep central portion) was defined exactly the same as the HE-DS. In contrast, conventional histopathology defines anterior and posterior margins based on the number of tumor-free HE-images multiplied by the average tissue block thickness (typically 3-5 mm). However, the actual distance between HE-images may vary, especially when they originate from sections taken from the outermost distal or proximal parts of the tissue block. Hence, spacing may vary between 1-10 mm. Secondly, the colormap that was used to highlight the inadequate margins on the MR- and HE-DS led to the interpretation of the inadequate margin as an area, rather than a point, which contrasts with conventional histopathology. Thirdly, the imaging methods and histopathological processing inherently introduce forces that can cause deformation or tearing of the resection specimen. During scanning, the deep central margin may be compressed against the support of the specimen, leading to an underestimation of the margin distance. Moreover, histopathological processing can cause rupturing (e.g., case 9) and shrinkage of the specimen. Although the effect of shrinkage might be compensated by the rigid scaling that was applied during registration, shrinkage might not be uniform over the entire specimen. For instance, Umstattd et al. (28) demonstrated that specimen shrinkage predominantly occurs in the healthy tissue rather than in the tumor, which might have been the situation in case 2.

The fact that sensitivity of the MR-DS was higher than specificity may be caused by the fact that the range of tumor overestimation (95HD: 0.9 mm – 11.8 mm) was far larger than the range of underestimation (95HD: 0.0 mm – 1.7 mm, with one outlier at 5.3 mm). Radiologists’ tendency to overestimate tumor volume leads to a low PPV (i.e. relatively many false positives, few true positives) and a high NPV (i.e. relatively many true negatives, few false negatives). However, overestimating the tumor has a more favorable clinical impact than underestimation. Despite the increased likelihood of unnecessary intraoperative re-resection, surgeons retain the discretion to disregard the indication when resecting structures that would significantly impact the patient’s quality of life. At the same time, this approach increases the likelihood of a successful re-resection.

A challenge frequently encountered during ex-vivo intra-operative margin assessment of tongue cancer is the loss of the anatomical relationship between the inadequate margins and the wound bed (8). One might argue that a technique allowing in-vivo assessment (during the actual resection) is more favorable. At our institute, we investigated the application of intra-operative ultrasound during tongue cancer surgery (5). This technique enabled us to scan the entire resection specimen, both in-vivo and ex-vivo. Based on our experience (5) and that of others (14, 29–32) in-vivo ultrasound can significantly enhance surgical resection margins. However, the diagnostic accuracy of the ultrasound in locating inadequate margins ex-vivo was moderate (area under the curve: 0.63). Additionally, the field of view is limited by the probe’s size and its acoustic penetration depth. Most other techniques for in-vivo assessment primarily reveal only the tumor’s mucosal extent (9, 11, 13). As no superior in-vivo technique has emerged thus far, ex-vivo margin assessment remains crucial for margin control. The fact that MR-DSs could be projected on screens in the surgical room may facilitate a clearer understanding of the relationship between the inadequate margin in the specimen and the wound bed.

Bekedam et al. (18) attempted to develop an ultrasound-based digital specimen – with a colormap - of the resection specimen. This was achieved by stacking ultrasound images of which the reciprocal relation was determined using an electromagnetic tracker. They found that the interpretation of margins became easier. One notable limitation, however, was the limited image-quality produced by the 10 MHz probe. Additionally, they did not differentiate the mucosa in their digital specimen from the actual resection plane.

Several groups have conducted research on ex-vivo MR of tongue cancer specimens. Steens et al. (22) evaluated the visibility of tongue cancer and resection margins from ten MR scans of resection specimens using a similar small-bore 7T MR as employed in our study. In three out of the ten specimens, the tumor was not visible, potentially due to a small depth of invasion (DOI), i.e. <1 mm.

In another study, Heidkamp et al. (23) studied ten tongue cancer specimens using a 3T MR scanner, situated in a surgical room, to improve logistics. The consequence of the lower field strength and larger bore was that the lower signal to noise ratio hampered the visibility of the transition between mucosa and resection plane. In our study, only a few cases received a score lower than 3 for the visibility of this transition. In case 3, the low visibility may have been attributed to the specimen being pressed against the cylindrical container. In case 5, it may have been caused by mucosal damage. Nevertheless, transition visibility did not seem to influence diagnostic accuracy significantly.

Giannitto et al. (24) performed a study with some similarities to our methods, i.e. they used a 3D-printed model of the tongue and tumor on which the resection specimen was attached for orientation of the specimen. Their 1.5T clinical ex-vivo MR-images showed a perfect diagnostic accuracy in predicting margin status (sensitivity and specificity both 100%). However, they stated their results were inconclusive due to the small sample size (n=10) and relatively high number of true negatives.

Several limitations of our study need to be acknowledged. Firstly, the sample size was small, which implies that our results cannot be conclusive. Secondly, despite the efforts to align HE- with MR-images, minor inaccuracies might have been introduced (as shown in **Supplementary Data**). Thirdly, the specimen’s outlines, derived from the MR-images, were utilized to reconstruct the resection plane for both the MR- and HE-DSs. This implies that margin underestimation resulting from compression is reflected in the HE-DS but not in the outcomes of conventional histopathological analysis. Consequently, the HE-DS yields different results when compared to the established clinical standard, raising questions about its accuracy as a reference standard. Finally, the radiologists are not specifically trained in tumor delineation on MR-images of the resection specimens. As a result, their performance during this study might have been influenced by a learning curve.

Future studies should consider a larger sample size to strengthen the validity of the results. Other MR contrasts, such as diffusion or enhanced T2 contrast on the T2W TSE sequence used in this study, should be explored to optimize the visibility of the tumor and mucosa. Efforts to improve registration accuracy, such as setups that control the spacing between HE-images, are currently being pursued. Radiologists should be provided with a training set to optimize their outline performances on MR-images and improve their inter-observer agreement (especially for detecting the mucosa). Meanwhile, such a training set can also be used in deep learning to train a convolutional neural network capable of automatically outline tumors from healthy tissue (33). By saving a vast amount of time, as manual outline is a time-consuming effort, deep learning may optimize the challenging logistics faced when ex-vivo MR is used in clinical practice. At the time of writing, such a training set is currently under construction.

## Conclusion

In conclusion, this proof of concept for volumetric assessment of resection margins using MR-DSs, as determined by radiologists, demonstrates promising potential for further development. This approach can enhance our understanding of the position of inadequate margins within the resection specimen. In the near future, this method could assist surgeons during tongue cancer resections by guiding them toward more precise and adequate direct intraoperative re-resection. Future studies should prioritize establishing a reliable registration with histopathology to validate the MR-DS. This step is crucial, especially if these models can be utilized in creating training sets for deep learning applications. Nonetheless, our study offers a foundational proof of principle, paving the way for subsequent studies to validate, apply, and refine this technique further toward clinical implementation.

## Data availability statement

The raw data supporting the conclusions of this article will be made available by the authors, after reasonable request.

## Ethics statement

The requirement of ethical approval was waived by METC NedMec, University Medical Center Utrecht for the studies involving humans because the study was not a subject to the Dutch Medical Research Involving Human Subjects Act (WMO), since the particpants were no subject to procedures or were not required to follow rules of behaviour (please consult https://english.ccmo.nl/investigators/legal-framework-for-medical-scientific-research/your-research-is-it-subject-to-the-wmo-or-not). The studies were conducted in accordance with the local legislation and institutional requirements. Written informed consent for participation in this study was provided by the participants’ legal guardians/next of kin. Written informed consent was obtained from the individual(s) for the publication of any potentially identifiable images or data included in this article.

## Author contributions

KK: Conceptualization, Data curation, Formal analysis, Investigation, Methodology, Project administration, Software, Validation, Visualization, Writing – original draft. JD: Formal Analysis, Investigation, Writing – review & editing. BK: Investigation, Writing – review & editing. KW: Methodology, Resources, Visualization, Writing – review & editing. AT: Methodology, Resources, Visualization, Writing – review & editing. GB: Investigation, Methodology, Writing – review & editing. Rv: Supervision, Writing – review & editing. RB: Supervision, Writing – review & editing. RN: Funding acquisition, Supervision, Writing – review & editing. MP: Funding acquisition, Resources, Supervision, Visualization, Writing – review & editing.

## References

[1] National Cancer Institute. SEER Cancer Stat Facts: Tongue Cancer(2023). Available online at: https://seer.cancer.gov/statfacts/html/tongue.html.

[2] Liao CT Chang JTC Wang HM Ng SH Hsueh C Lee LY. Analysis of risk factors of predictive local tumor control in oral cavity cancer. Ann Surg Oncol. (2008) 15:915–22. doi: 10.1245/s10434-007-9761-5 18165878

[3] Helliwell T Woolgar J. Standards and datasets for reporting cancers(2013). Available online at: www.nice.org.uk/accreditation.

[4] Smits RWH Koljenović S Hardillo JA Ten Hove I Meeuwis CA Sewnaik A. Rection margins in oral cancer surgery: room for improvementRes. Head Neck. (2016) 38:E2197–203. doi: 10.1002/hed.24075 25899524

[5] de Koning KJ van Es RJJ Klijn RJ Breimer GE Willem Dankbaar J Braunius WW. Application and accuracy of ultrasound-guided resections of tongue cancer. Oral Oncol. (2022) 133:1–8. doi: 10.1016/j.oraloncology.2022.106023 35901543

[6] Yang ZH Chen WL Huang HZ Bin PC Li JS. Quality of life of patients with tongue cancer 1 year after surgery. J Oral Maxillofac Surgery. (2010) 68:2164–8. doi: 10.1016/j.joms.2009.09.048 20542366

[7] Jehn P Stier R Tavassol F Dittmann J Zimmerer R Gellrich NC. Physical and psychological impairments associated with mucositis after oral cancer treatment and their impact on quality of life. Oncol Res Treat. (2019) 42:342–8. doi: 10.1159/000499720 30970370

[8] Kubik MW Sridharan S Varvares MA Zandberg DP Skinner HD Seethala RR. Intraoperative margin assessment in head and neck cancer: A case of misuse and abuse? Head Neck Pathol. (2020) 14:291–302. doi: 10.1007/s12105-019-01121-2 32124417 PMC7235105

[9] Tirelli G Piovesana M Marcuzzo AV Gatto A Biasotto M Bussani R. Tailored resections in oral and oropharyngeal cancer using narrow band imaging. Am J Otolaryngol – Head Neck Med Surg. (2018) 39:197–203. doi: 10.1016/j.amjoto.2017.11.004 29150027

[10] Durham JS Brasher P Anderson DW Yoo J Hart R Dort JC. Effect of fluorescence visualization-guided surgery on local recurrence of oral squamous cell carcinoma: A randomized clinical trial. JAMA Otolaryngol Head Neck Surg. (2020) 146:1149–55. doi: 10.1001/jamaoto.2020.3147 PMC7545352 33034628

[11] Algadi HH Abou-Bakr AAE Jamali OM Fathy LM. Toluidine blue versus frozen section for assessment of mucosal tumor margins in oral squamous cell carcinoma. BMC Cancer. (2020) 20:1–8. doi: 10.1186/s12885-020-07644-0 PMC7691066 33238944

[12] Voskuil FJ de Jongh SJ Hooghiemstra WTR Linssen MD Steinkamp PJ de Visscher SAHJ. Fluorescence-guided imaging for resection margin evaluation in head and neck cancer patients using cetuximab-800CW: A quantitative dose-escalation study. Theranostics. (2020) 10:3994–4005. doi: 10.7150/thno.43227 32226534 PMC7086353

[13] Umeda M Shigeta T Takahashi H Minamikawa T Komatsubara H Oguni A. Clinical evaluation of Lugol’s iodine staining in the treatment of stage I-II squamous cell carcinoma of the tongue. Int J Oral Maxillofac Surg. (2011) 40:593–6. doi: 10.1016/j.ijom.2010.11.026 21334851

[14] Tarabichi O Kanumuri V Juliano AF Faquin WC Cunnane ME Varvares MA. Intraoperative ultrasound in oral tongue cancer resection: feasibility study and early outcomes. Otolaryngol – Head Neck Surg (United States). (2018) 158:645–8. doi: 10.1177/0194599817742856 29161194

[15] Barroso EM ten Hove I Bakker Schut TC Mast H van Lanschot CGF Smits RWH. Raman spectroscopy for assessment of bone resection margins in mandibulectomy for oral cavity squamous cell carcinoma. Eur J Cancer. (2018) 92:77–87. doi: 10.1016/j.ejca.2018.01.068 29428867

[16] Van Keulen S Nishio N Birkeland A Fakurnejad S Martin B Forouzanfar T. The sentinel margin: Intraoperative ex vivo specimen mapping using relative fluorescence intensity. Clin Cancer Res. (2019) 25:4656–62. doi: 10.1158/1078-0432.CCR-19-0319 PMC7021202 31142505

[17] Brouwer de Koning SG Karakullukcu MB Lange CAH Schreuder WH Karssemakers LHE Ruers TJM. Ultrasound aids in intraoperative assessment of deep resection margins of squamous cell carcinoma of the tongue. Br J Oral Maxillofac Surg. (2020) 58:285–90. doi: 10.1016/j.bjoms.2019.11.013 32044145

[18] Bekedam NM Smit JN de Koekkoek – Doll PK van Alphen MJA van Veen RLP Karssemakers LHE. Intra-operative resection margin model of tongue carcinoma using 3D reconstructed ultrasound. Adv Oral Maxillofac Surg. (2021) 4:100154. doi: 10.1016/j.adoms.2021.100154

[19] Steinkamp PJ Voskuil FJ van der Vegt B Doff JJ Schepman KP de Visscher SAHJ. A standardized framework for fluorescence-guided margin assessment for head and neck cancer using a tumor acidosis sensitive optical imaging agent. Mol Imaging Biol. (2021) 23:809–17. doi: 10.1007/s11307-021-01614-z PMC8578180 34031845

[20] Du E Ow TJ Lo YT Gersten A Schiff BA Tassler AB. Refining the utility and role of Frozen section in head and neck squamous cell carcinoma resection. Laryngoscope. (2016) 126:1768–75. doi: 10.1002/lary.25899 27113207

[21] Long SM Mclean T Valero Mayor C Fitzgerald CWR Feit NZ Katabi N. Use of intraoperative frozen section to assess final tumor margin status in patients undergoing surgery for oral cavity squamous cell carcinoma. JAMA Otolaryngol Head Neck Surg. (2022) 148:1–7. doi: 10.1001/jamaoto.2022.2131 PMC9353701 35925571

[22] Steens SCA Bekers EM Weijs WLJ Litjens GJS Veltien A Maat A. Evaluation of tongue squamous cell carcinoma resection margins using ex-vivo MR. Int J Comput Assist Radiol Surg. (2017) 12:821–8. doi: 10.1007/s11548-017-1524-6 PMC5420007 28130702

[23] Heidkamp J Weijs WLJ van Engen-van Grunsven ACH de Laak-de Vries I Maas MC Rovers MM. Assessment of surgical tumor-free resection margins in fresh squamous-cell carcinoma resection specimens of the tongue using a clinical MRI system. Head Neck. (2020) 42:2039–49. doi: 10.1002/hed.26125 PMC7496932 32119170

[24] Giannitto C Mercante G Disconzi L Boroni R Casiraghi E Canzano F. Frozen section analysis and real-time magnetic resonance imaging of surgical specimen oriented on 3D printed tongue model to assess surgical margins in oral tongue carcinoma: preliminary results. Front Oncol. (2021) 11:11. doi: 10.3389/fonc.2021.735002 PMC8698483 34956865

[25] Stathonikos N Nguyen TQ Spoto CP Verdaasdonk MAM van Diest PJ. Being fully digital: perspective of a Dutch academic pathology laboratory. Histopathology. (2019) 75:621–35. doi: 10.1111/his.13953 PMC6856836 31301690

[26] Bol GH Kotte ANTJ van der Heide UA Lagendijk JJW. Simultaneous multi-modality ROI delineation in clinical practice. Comput Methods Programs BioMed. (2009) 96:133–40. doi: 10.1016/j.cmpb.2009.04.008 19443076

[27] Caldas-Magalhaes J Kasperts N Kooij N Van Den Berg CAT Terhaard CHJ Raaijmakers CPJ. Validation of imaging with pathology in laryngeal cancer: Accuracy of the registration methodology. Int J Radiat Oncol Biol Phys. (2012) 82:e289–e298. doi: 10.1016/j.ijrobp.2011.05.004 21719209

[28] Umstattd LA Mills JC Critchlow WA Renner GJ Zitsch RP III. Shrinkage in oral squamous cell carcinoma: An analysis of tumor and margin measurements in *vivo*, post-resection, and post-formalin fixation. Am J Otolaryngol. (2017) 38:660–2. doi: 10.1016/j.amjoto.2017.08.011 28917966

[29] Adriaansens CMEM de Koning KJ de Bree R Dankbaar JW Breimer GE van Es RJJ. Ultrasound-guided resection for squamous cell carcinoma of the buccal mucosa: A feasibility study. Head Neck. (2023) 45:647–57. doi: 10.1002/hed.27281 PMC10107760 36528853

[30] Helbig M Flechtenmacher C Hansmann J Dietz A Tasman AJ. Intraoperative B-mode endosonography of tongue carcinoma. Head Neck. (2001) 23:233–7. doi: 10.1002/(ISSN)1097-0347 11428455

[31] Baek CH Son YI Jeong HS Chung MK Park KN Ko YH. Intraoral sonography-assisted resection of T1-2 tongue cancer for adequate deep resection. Otolaryngol – Head Neck Surg. (2008) 139:805–10. doi: 10.1016/j.otohns.2008.09.017 19041507

[32] Bulbul MG Tarabichi O Parikh AS Yoon BC Juliano A Sadow PM. The utility of intra-oral ultrasound in improving deep margin clearance of oral tongue cancer resections. Oral Oncol. (2021) 122:122. doi: 10.1016/j.oraloncology.2021.105512 PMC9375962 34564016

[33] Halicek M Dormer JD Little JV Chen AY Myers L Sumer BD. Hyperspectral imaging of head and neck squamous cell carcinoma for cancer margin detection in surgical specimens from 102 patients using deep learning. Cancers (Basel). (2019) 11:1–16. doi: 10.3390/cancers11091367 PMC6769839 31540063

