# Supplementary figures and images for: Feasibility of an MR-based digital specimen for tongue cancer resection specimens: a novel approach for margin evaluation
Source: Front Oncol. 2024 Mar 28;14:1342857. doi: 10.3389/fonc.2024.1342857 (PMC11007136; doi:10.3389/fonc.2024.1342857)

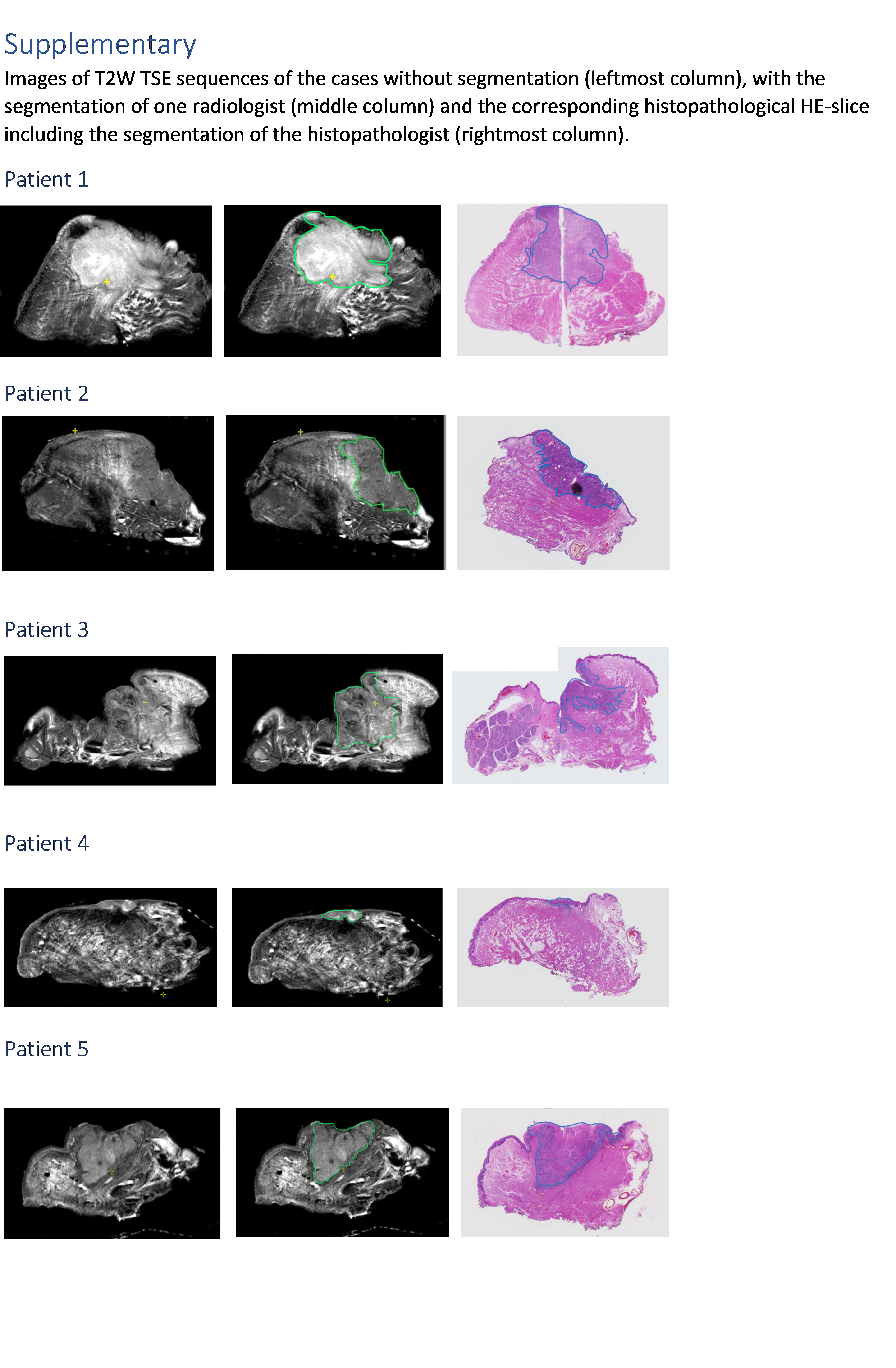

Supplement: Supplementary file 1 [file Image_1.tif]

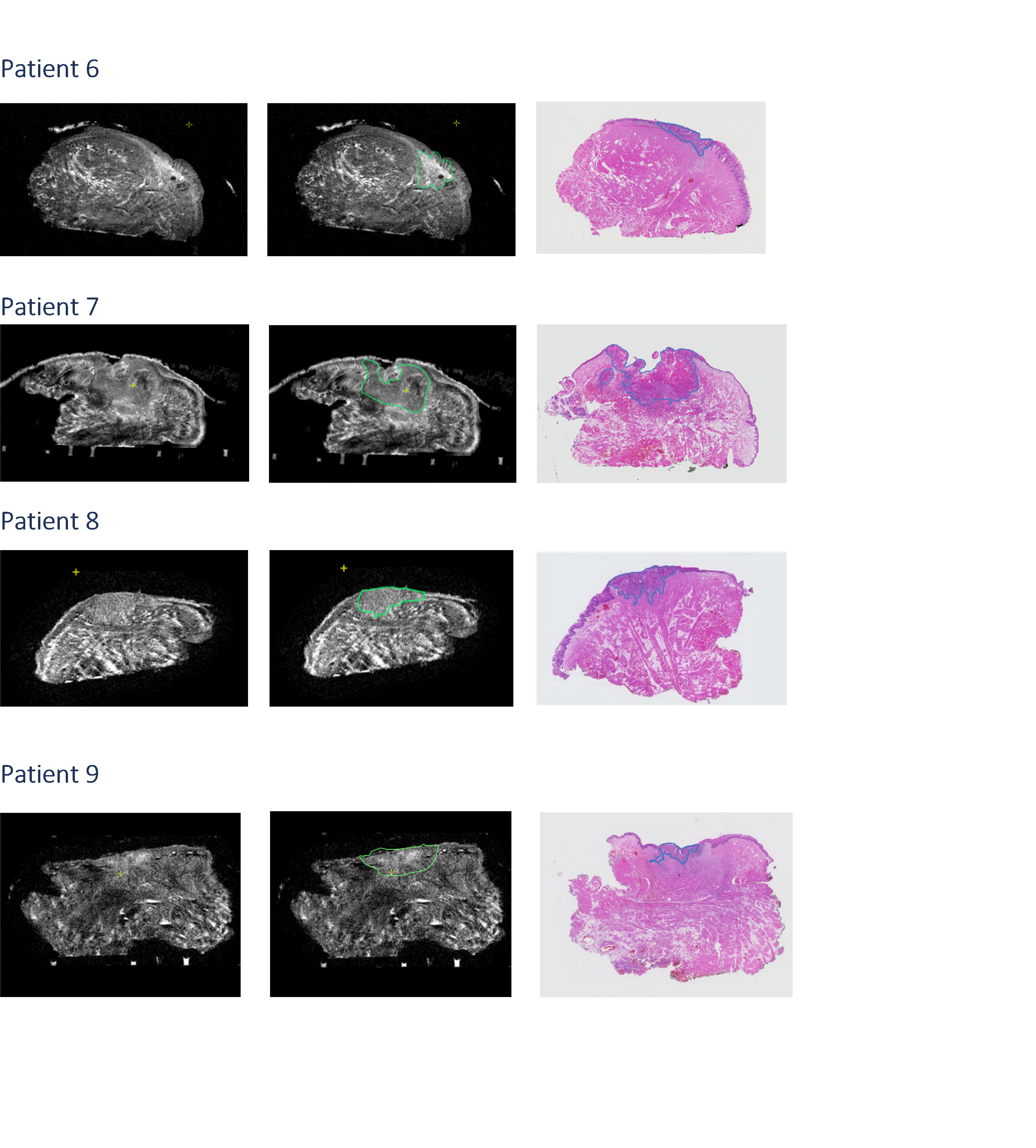

Supplement: Supplementary file 2 [file Image_2.tif]
